# Repeat GammaTile Brachytherapy in the Long-Term Management of Recurrent High-Grade Glioma: A Case Report

**DOI:** 10.7759/cureus.75053

**Published:** 2024-12-03

**Authors:** Jeff F Zhang, Michael D Mix, Anna Shapiro, Harish Babu

**Affiliations:** 1 Neurological Surgery, Upstate University Hospital, Syracuse, USA; 2 Radiation Oncology, Upstate University Hospital, Syracuse, USA

**Keywords:** cns tumors, gammatile, intracranial brachytherapy, primary brian tumor, recurrent high-grade glioma

## Abstract

Over the past two decades, despite the emergence of various novel therapies for glioblastoma, patient survival outcomes remain poor, particularly in the recurrent stage of the disease. Cesium-131 (Cs-131) brachytherapy presents a promising treatment option for patients with newly diagnosed and recurrent brain neoplasms, enabling the initiation of radiation therapy at the time of tumor resection. This approach eliminates the typical delay in therapy following surgery and the need for multiple return visits for fractionated external beam radiotherapy. This report describes a patient who underwent repeat Cs-131 brachytherapy implantations in the surgical cavity following the resection of a recurrent glioblastoma, achieving a good quality of life and survival of over 36 months.

## Introduction

Glioblastoma is the most common primary malignant brain tumor, accounting for 48.6% of all malignant central nervous system tumors [1]. Despite advancements in treatment over the past two decades, patient outcomes remain dismal, with a median overall survival (OS) of only 15 months following initial diagnosis [2]. The standard treatment for glioblastoma includes maximal safe resection followed by radiotherapy with concomitant temozolomide [3]. Typically, external beam radiotherapy (EBRT) is administered five days a week over six weeks, delivering a total dose of 60 Gy in 30 fractions. However, even with aggressive adjuvant chemoradiotherapy, tumor recurrence is nearly universal. Salvage treatment options for recurrent disease remain limited, with bevacizumab being the primary standard option.

Approximately 85% of recurrences occur within the margins of the prior resection cavity [4], making intracavitary adjuvant brachytherapy an attractive strategy. This approach offers potential advantages, including the immediate initiation of radiation therapy, the elimination of multiple visits required for EBRT, and intensified radiation delivery to areas most prone to disease progression.

Intracranial brachytherapy has been previously investigated using iodine-125 (I-125), but it was associated with high rates of adverse radiation effects (14-33%), likely due to I-125’s long half-life of 59.4 days [5]. GammaTile^®^ (GT) brachytherapy, incorporating Cesium-131 (Cs-131), may provide a better therapeutic ratio due to Cs-131’s shorter half-life of 9.7 days [6] and higher dose rate of 0.342 Gy/hr [7]. GT received FDA approval in 2018 for recurrent brain tumors and in 2020 for newly diagnosed malignant brain tumors [8].

GT utilizes a bioresorbable collagen matrix embedded with four Cs-131 seeds. This design allows for precise dosimetry and delivers 88% of the therapeutic dose (80-120 Gy to the first few millimeters from the implantation site) within 30 days, and over 95% within six weeks (equivalent to four half-lives of Cs-131), during which the collagen matrix fully dissolves [9]. This fixed-seed configuration offers more predictable radiation distribution compared to the earlier “loose-seed” implantation methods.

While GT appears to reduce the incidence of adverse radiation effects [10,11], its impact on improving progression-free survival and OS in recurrent glioblastoma patients remains an active area of investigation. Furthermore, survival outcomes in patients treated with repeat brachytherapy are rarely reported in the literature.

This report presents a case of two separate GT implantations in the management of multiple recurrent glioblastoma. The patient achieved more than three years of survival from the initial diagnosis while maintaining good functional status, without evident treatment-related toxicity or surgical wound complications.

## Case presentation

**Figure 1:**
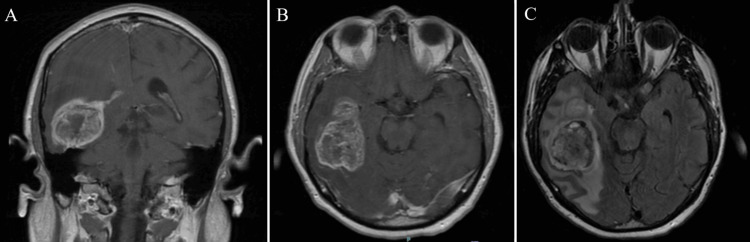
Initial imaging at the time of diagnosis: (A) Coronal and (B) axial T1-weighted MRI with contrast and (C) FLAIR sequence showing a large right temporal mass with significant vasogenic edema

**Figure 2:**
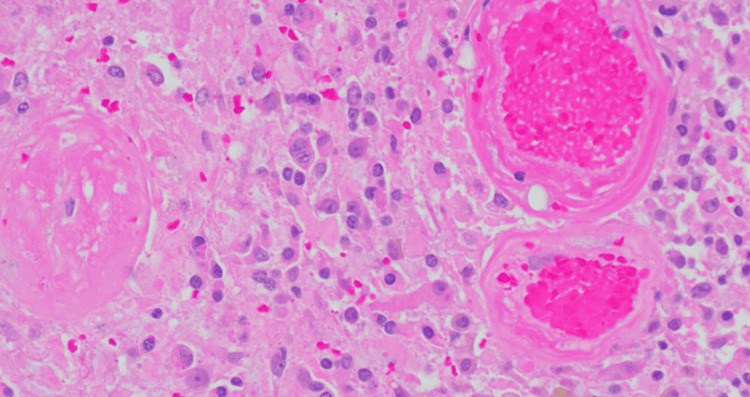
Histological slide of the resected tumor tissue The image shows neoplastic cells with ovoid, enlarged, hyperchromatic nuclei and variable eosinophilic fibrillar cytoplasm. Immunohistochemical staining revealed GFAP positivity and a Ki67 proliferation index estimated at approximately 10%.

The patient was a 62-year-old right-handed male who presented to the emergency department of an outside hospital with several weeks of worsening headaches and gait instability. MRI with and without contrast revealed a large right temporal mass accompanied by significant vasogenic edema (Figure 1). During that hospital admission in November 2020, the patient underwent partial resection of the mass with Gliadel wafer implantation. Histopathological analysis of the tumor sample (Figure 2) confirmed a high-grade glioma consistent with glioblastoma, characterized as IDH-wild type, MGMT-unmethylated, and BRAF V600E-mutated. Following the resection, the patient received standard-of-care treatment at our institution, consisting of temozolomide chemotherapy (140 mg) in combination with radiation therapy (60 Gy in 30 fractions).

**Figure 3:**
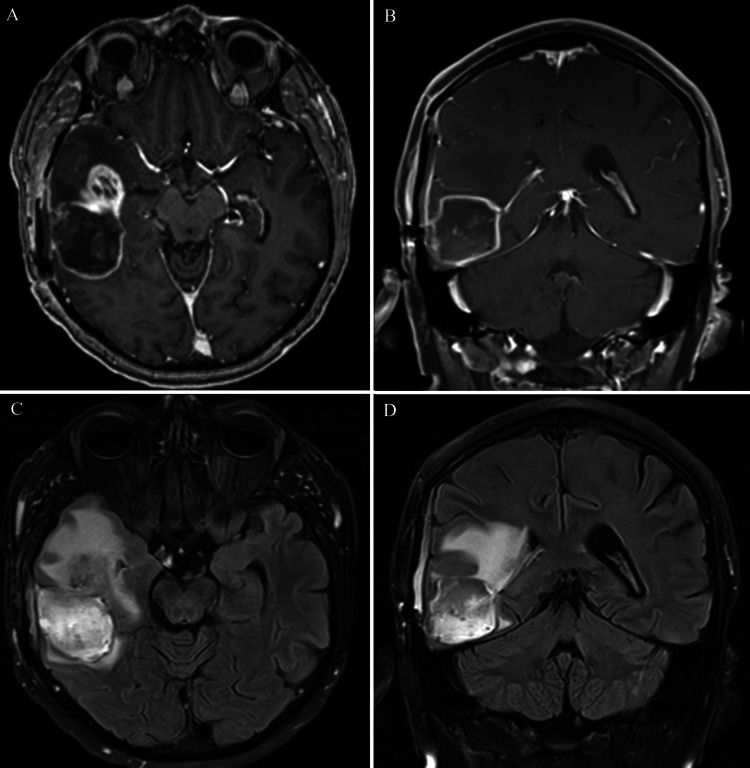
Axial (A) and coronal (B) T1-weighted MRI with contrast, along with axial (C) and coronal (D) FLAIR sequences, demonstrating tumor recurrence

**Figure 4:**
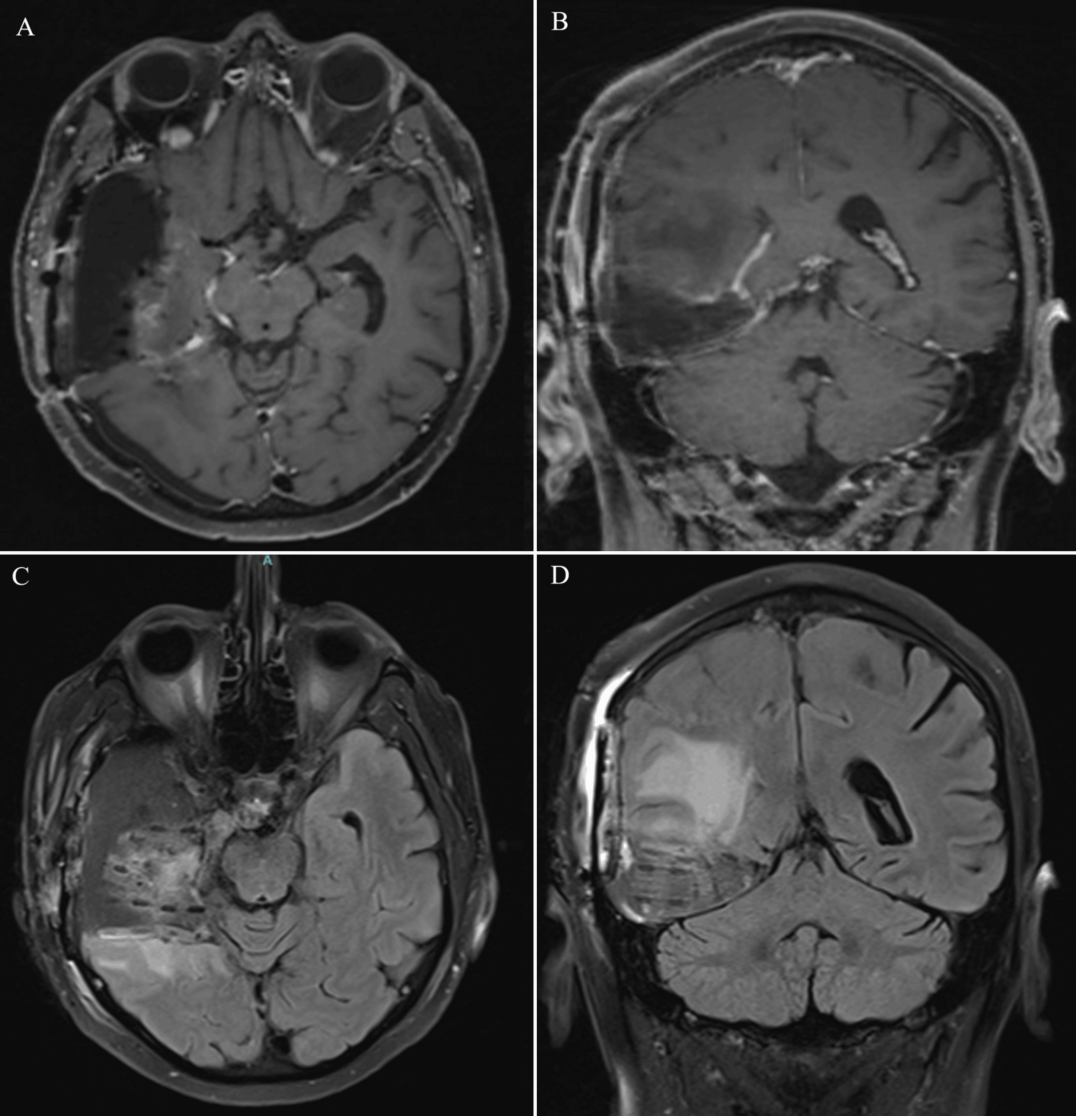
Postsurgical imaging after the patient’s second resection and first GT implant placement for tumor recurrence: (A) Axial and (B) coronal T1-weighted MRI with contrast, along with (C) axial and (D) coronal FLAIR sequences, demonstrating postoperative changes and implant placement GT, GammaTile

**Figure 5:**
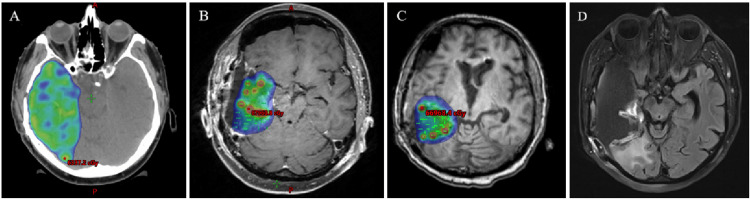
Dosimetric images with 60 Gy isodose clouds illustrating radiation coverage for (A) adjuvant radiotherapy after the initial resection, (B) first GT placement following the second resection, and (C) second GT placement following the third resection. (D) MRI FLAIR sequence showing the relative locations of GT placements after the second implantation GT, GammaTile

Four months after the initial resection and following one cycle of pembrolizumab, the patient presented with blurry vision, and MRI confirmed tumor recurrence (Figure 3). Given his relatively good performance status, the patient was offered and underwent repeat resection, with placement of seven GTs and a prescribed dose of 60 Gy to a depth of 0.5 cm. Postoperative MRI (Figure 4) showed near-total resection of the previously noted enhancing lesion. Representative dosimetric images are presented in Figure 5.

The patient was discharged home a few days later without postoperative complications or the need for steroids. Final surgical pathology confirmed a diagnosis of recurrent glioblastoma. However, three days after discharge, the patient returned to the emergency department with low-grade fevers and fatigue. A CT head was negative for acute findings. The patient was discharged with symptomatic management and presented to the clinic three days later with persistent fevers, mild headache, and neck pain. Lumbar puncture revealed a normal opening pressure, but elevated protein and white blood cell count (total nucleated cells of 1,159 per µL, with normal percentages of neutrophils and lymphocytes) and decreased glucose. A diagnosis of aseptic meningitis was made, and the patient was treated with dexamethasone, resulting in symptomatic improvement. The patient subsequently restarted temozolomide at a dose of 380 mg daily.

**Figure 6:**
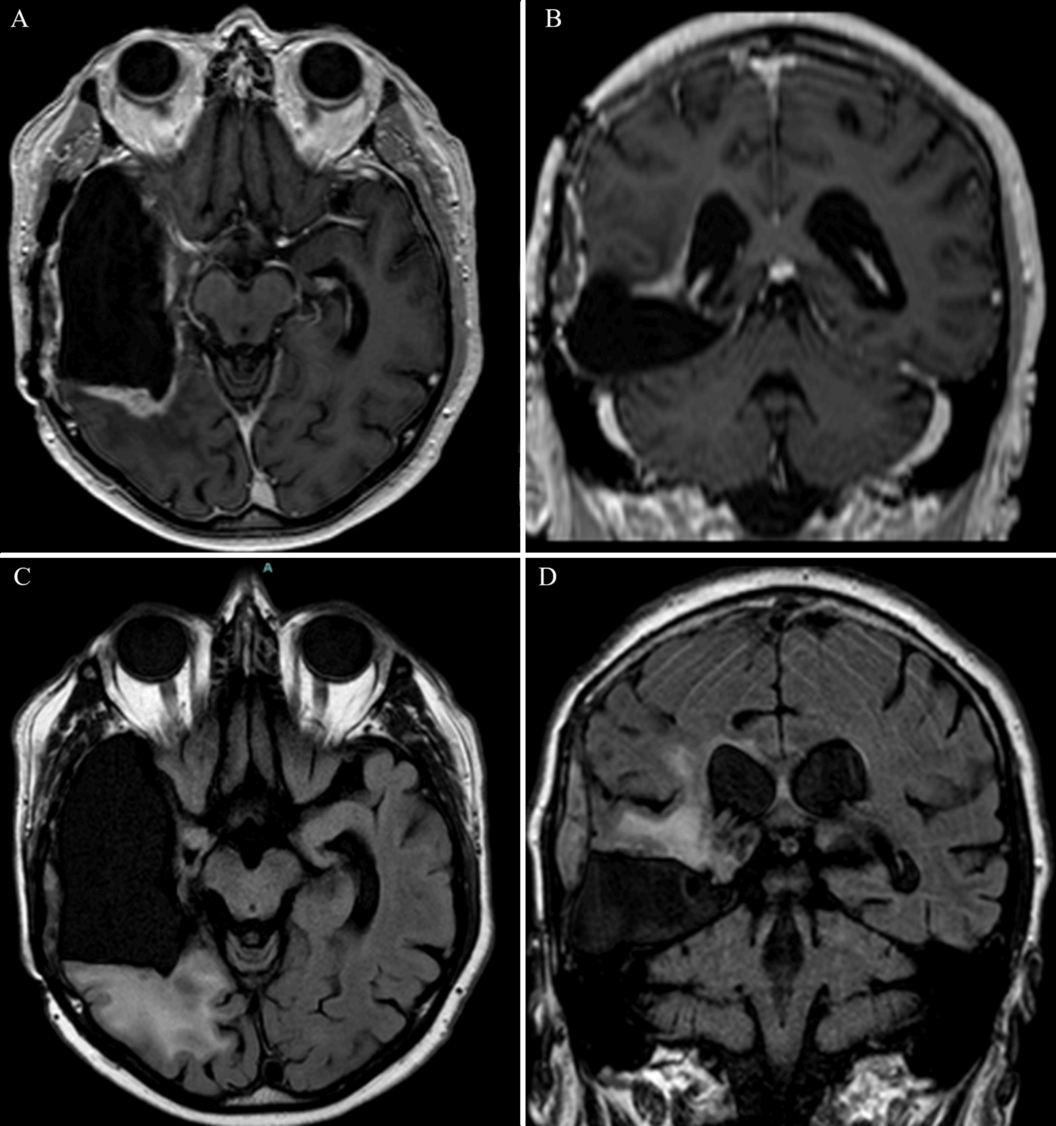
Axial (A) and coronal (B) T1-weighted MRI with contrast, along with axial (C) and coronal (D) FLAIR sequences at the time of second recurrence, showing increased enhancement along the posterior aspect of the patient’s resection cavity

The patient’s clinical status and surveillance MRIs remained stable until December 2022 (25 months following the initial resection), during which time temozolomide had been discontinued. The patient then reported a new left-sided peripheral visual field deficit, and MRI findings (Figure 6) were consistent with tumor progression. A third resection was performed, followed by a second GT implantation involving eight tiles, with a prescribed dose of 60 Gy to a depth of 0.5 cm. A cumulative dose, including external beam treatment and the two GT implants, was calculated in the treatment planning system. The optic apparatus (both nerves and chiasm) received a maximum point dose of 81 Gy, with a 0.1 cc dose of 74 Gy. The brainstem received a maximum point dose of 141 Gy, and the doses of 0.1 cc, 1 cc, and 10 cc were 133 Gy, 114 Gy, and 49 Gy, respectively. These accumulated doses represent algebraic sums, with no correction for biologically equivalent doses.

Surgical pathology was consistent with a recurrent tumor admixed with radiation necrosis. The patient was discharged home two days later without complications and reported visual improvement during a follow-up clinic visit two days after discharge.

**Figure 7:**
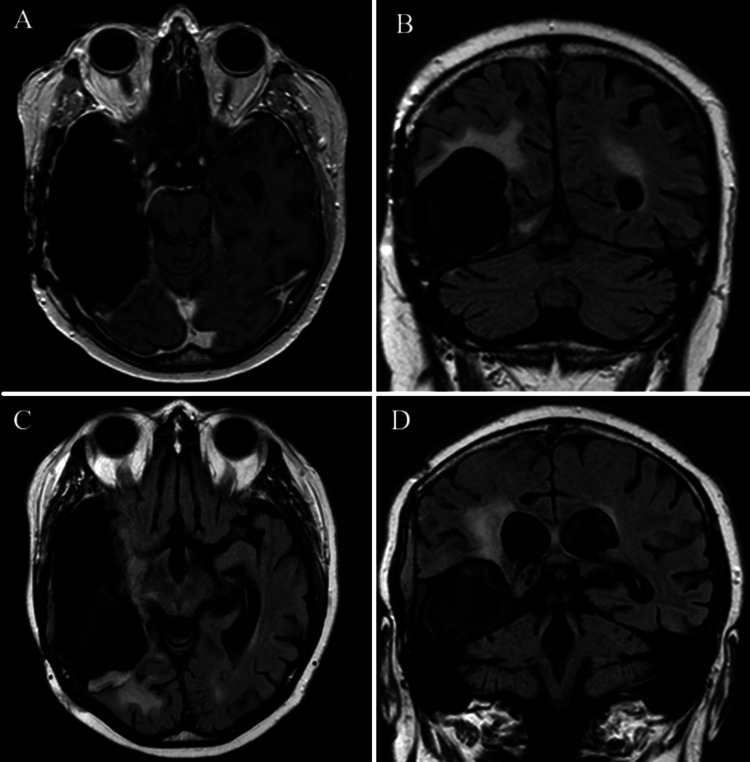
Axial (A) and coronal (B) T1-weighted MRI with contrast, along with axial (C) and coronal (D) FLAIR sequences from the patient’s last recorded MRI, showing enhancement along the posterior and lateral margins of the prior resection cavity

Vemurafenib was initially started but discontinued shortly afterward due to poor tolerance and negative BRAF V600E testing on the updated surgical specimen. The patient was then started on bevacizumab and lomustine approximately three months following his third resection. The patient was later hospitalized for a perforated colon and entered a period of observation, during which chemotherapy treatment was paused. A surveillance MRI in December 2023 (37 months following the initial resection) showed enhancement along the posterior wall and right ventricular margin of the prior resection cavity (Figure 7). At the time of the last follow-up in our clinic, the patient had a good neurological status but elected to enter hospice care in January 2024 due to his refusal to undergo further abdominal surgery.

## Discussion

The use of brachytherapy in glioblastoma patients presents a potential alternative or complement to existing therapeutic approaches, such as EBRT and radiosurgery, particularly in cases of recurrent disease. To our knowledge, this report represents the first published case of a patient treated with two separate GT implants. The patient survived for 10 months following the second brachytherapy treatment and approximately 37 months from the initial diagnosis.

Brachytherapy has an established role in the treatment of other malignancies, such as cervical, uterine, and prostate cancers, among others [9]. However, its effectiveness in treating intracranial neoplasms remains less clear. Two prior randomized controlled trials did not show significant survival benefits when I-125 implants were combined with EBRT [12] or with EBRT and carmustine [13] in patients with high-grade glioma. These trials were conducted before the routine use of neuronavigation to aid in achieving gross-total resection and before the widespread use of sensitizing chemotherapy [8]. Additionally, there may be advantages to using Cesium-containing GT compared to traditional iodine-based seeds, given its higher dose rates and more predictable dosimetry.

While GT has shown promise in treating recurrent meningiomas [8,14] and metastatic brain tumors [8,15], its use in glioblastoma cases is less well documented. In a study of 22 patients with IDH-wild type recurrent glioblastoma treated with GT, the median OS for the treatment group was 24.4 months, compared to 17 months in the control group who did not receive GT implantation (p = 0.0063) [16]. Importantly, the study reported no differences in length of hospital stay or postoperative complication rates between the two groups [16]. Another report involving 20 patients with recurrent glioblastoma found the median post-recurrence OS to be nine months for those treated with Cs-131, with no cases of radiation necrosis in patients treated concurrently with bevacizumab [11]. However, it is important to recognize that the patients eligible for intensified therapy may have had clinical characteristics that predisposed them to improved survival, and thus, selection bias must be considered when interpreting these results. Ongoing trials are expected to provide further insight into the role of GT in the treatment of newly diagnosed and recurrent glioblastoma. Two randomized trials, the GammaTile and Stupp in Newly Diagnosed GBM (GESTALT) trial and NCT04427384, are currently enrolling patients.

The eventual clinical decline of our patient was likely multifactorial, including the challenges of multiple recurrences requiring repeat resections and other health complications, which ultimately led to the family’s decision to pursue hospice care. Nonetheless, survival of 37 months in a case of MGMT-unmethylated glioblastoma is significantly above the expected median survival for this patient population. Further research is needed to better understand the role of GT brachytherapy in recurrent high-grade glioma and whether repeat implantation is a safe and viable treatment option.

## Conclusions

Despite advances in chemotherapy, EBRT, and tumor-treating fields over the past two decades, outcomes for patients with glioblastoma remain poor, and there is currently no established standard of care for recurrent disease. Repeat resection and GT brachytherapy offer a potential treatment option for patients with recurrent high-grade glioma, and studies are ongoing to evaluate its use in newly diagnosed glioblastoma. This report highlights a case in which a patient diagnosed with glioblastoma underwent two separate GT implantations during their treatment course and survived for 37 months without evident treatment-related toxicity.
